# Temporal resolution: assessment procedures and parameters for school-aged children

**DOI:** 10.5935/1808-8694.20130057

**Published:** 2015-10-04

**Authors:** Maria Isabel Ramos do Amaral, Paula Maria Faria Martins, Maria Francisca Colella-Santos

**Affiliations:** a Speech and Hearing Therapist. PhD Student in Children and Adolescent Health - CIPED/FCM/UNICAMP (Professor at the Department of Speech and Hearing Therapy of the State University of the Midwest - UNICENTRO/Irat-PR).; b MSc in Children and Adolescent Health - CIPED/FCM/UNICAMP (Clinical Speech and Hearing Therapist).; c Speech and Hearing Therapist. PhD in Human Communication Disorders - UNIFESP/EPM (Professor and Coordinator - Speech and Hearing Therapy Program - School of Medical Sciences - University of Campinas (UNICAMP).

**Keywords:** auditory perception, child, hearing tests

## Abstract

Temporal resolution enables the identification of fine differences in speech segmental aspects. Random Gap Detection Test (RGDT) and Gaps-In-Noise Test (GIN) evaluate such skills, by using different acoustic parameters.

**Objective:**

To compare the performance of normal school aged children without learning disabilities and/or hearing complaints in the GIN and RGDT, and analyze potential performance differences in these two procedures.

**Method:**

Cross sectional contemporary cohort study. 28 children, aged 8-10 years were evaluated. After peripheral audiological evaluation, RGDT and GIN were performed.

**Results:**

There were no statistical differences in performance between gender and age on the RGDT and GIN tests, between the right and left ears on the GIN test, and between frequencies on the RGDT test. The mean detection threshold gap for RGDT was 9.25 ± 3.67 ms, and for GIN was 4.32 ± 0.61 ms (right ear) and 4.43 ± 0.79 ms (left ear). The results of the GIN Test were statistically lower than those from the RGDT (*p* < 0.001).

**Conclusion:**

Both tests indicated normal temporal resolution for all 28 children. GIN test presents advantages regarding the ease of application, task variable, stimuli and presentations form. However, the RGDT has advantages concerning the time required for administration and scoring.

## INTRODUCTION

We know that the proper acquisition of language and speech is highly dependent on hearing, and such sense is seen as part of a specialized system of communication and involves much beyond the mere detention of an acoustic signal. Many neurophysiological and cognitive processes are necessary for the correct perception, recognition, decoding and interpretation of the auditory information and consequent learning^1^.

Studies prove that the skill involved in the temporal auditory processing of sounds, such as temporal ordering and resolution, are closely related with speech perception and suprasegmental traces. For a proper message decoding, the acoustic clues pertaining to frequency, intensity and time must be accurately processed by the Central Auditory Nervous System (CANS). Deficits in the ordering and resolution skills may lead to low school performance associated with changes in the reading, writing and learning processes^2^, ^3^.

Temporal resolution (TR) is defined as the necessary minimum time for the CANS to discriminate two acoustic stimuli. The ability of the auditory system to detect fast changes in the sound stimulus is an important factor in speech perception, because it helps in the identification of small phonetic elements present in speech, and alterations in this auditory ability suggest interference in normal speech perception and in the recognition of phonemes^4^, ^5^.

The simplest psychoacoustic methods used to evaluate TR are based on the detection of interstimuli time intervals, the so-called gaps, which goal is to establish the shortest gap interval perceived between two sounds (gap detection threshold). Today, we have two gap-detection-based TR tests available for clinical use: the Random Gap Detection Test (RGDT)^6^ and the Gaps-In-Noise (GIN)^7^ detection test.

Parameters in the two tests differ in relation to the duration of each gap, stimulus presentation mode and requested task. Some studies have been carried out to compare Brazilian young adults^8^, Brazilian school-aged children^9^ and North American school-aged children^10^ vis-à-vis performance in the RGDT. Despite the small number of individuals in each study, all concluded that both procedures are fit to identify the individuals considered normal vis-à-vis the TR skill; however, we found differences between the results pertaining to gender, frequency and type of skill required; and a significant difference in the comparative performance of the two tests.

In view of the recommendations from the American Speech-Language-Hearing Association^11^ regarding the importance of including TR assessment procedures in the battery of auditory processing tests, we need further studies to establish the pros and cons of each available procedure, especially in relation to their application in children. Such fact has been stressed by Balen et al.^9^, whose conclusion in their paper points to the real need for better understanding the skills involved in each one of the TR procedures, since it is still not possible to establish which of the two protocols would be better for assessing children in the clinical setting.

Since we do not have a consensus regarding which TR assessment method is more efficient or practical to be added to the assessments in clinical practice, this choice shall be made by the speech and hearing therapists. Perhaps for the fact that the RGDT is older than the GIN, it is more frequently chosen, although the papers cited highlight some advantages of the GIN in relation to its application and result calculations, as well as challenging that the GIN and the RGDT are not evaluating the same auditory skill or require different non-auditory processes in the requested tasks^8^, ^9^, ^10^.

The goal of this study was to assess the TR in a larger number of school-aged children, vis-à-vis what has been published in literature, in the age range between 8 and 10 years, without hearing complaints and/or school-aged children, using the RGDT and GIN tests, considering the male and female genders and right and left ears. The study aimed at checking the sample performance in each procedure, as well as comparing, analyzing and discussing the differences in the parameters utilized between the two methods of TR assessment, seeking to find contributions for the pediatric clinical practice.

## METHOD

Contemporary cohort study, carried out in the same institution where the research was made. Approved under protocol # 626/2007 from the Ethics in Research Committee. All the parents and/or guardians agreed to the participation in the study and had signed the Informed Consent Form.

The inclusion criteria were: age between 8 and 10 years, being a basic education student from the Public School Network of the city of Campinas, SP, and have Portuguese as first language. The exclusion criteria were: difficulties with language and/or learning; a past of neurological, psychiatric or ear disorders that could compromise hearing, such as recurrent and/or chronic otitis media.

Such criteria were established by means of a detailed medical interview with the parents and/or guardians, and also by means of a questionnaire given to the teachers to answer about the student's school performance and participation in class; as well as questions pertaining to the child's behavior and interaction at school.

The selected children were submitted to the following procedures: Basic audiological evaluation (ear canal inspection, pure tone audiometry, logoaudiometry and immittance audiometry, following the normality criteria proposed by Northern & Downs^12^ and Carvallo^13^), and a Simplified Auditory Processing Evaluation carried through according to the normality criteria from Pereira et al.^14^, including the digits dicotic test^15^.

Those participants who did not fit in some of these normality criteria were also taken off the sample; and were referred to a complete otorhinolaryngological assessment of their auditory processing.

After that, the children who passed were submitted to the two TR tests: The Random Gap Detection Test (RGDT)^6^ and the Gaps-In-Noise Test (GIN)^7^, which were recorded in a Compact Disc and applied by means of an Audiometer from Interacoustics - AC40, connected to a Phillips CD (Compact Disc) player, in an acoustic booth, presented at 50 dBSL (in accordance with the mean value of the 500, 1000 and 2000 Hz tonal auditory thresholds).

RGDT is considered a TR test, and consists of the binaural presentation of pairs of pure tones in the frequencies of 500, 1000, 2000 and 4000 Hz, with intervals (“gaps”) between the two tones that randomly increase or reduce in duration, varying between intervals of 0, 2, 5, 10, 15, 20, 25, 30 and 40 milliseconds (ms). The test presents a practice band which was carried out before the test onset.

The child was instructed to answer with gestures whether hearing one or two tones, that is, whether or not an interstimuli gap was noticed. The test's goal was to establish the shortest time interval between two pure tones that could be perceived by the patient, that is, to determine the gap detection threshold, which was individually calculated for each tested frequency, as well as to calculate the total test response, through the arithmetic mean of results in the four evaluated frequencies.

The GIN is also a TR test; however, of monaural presentation, made up of a practice band and four test bands, and we employed only test-band 1 and test-band 2 (one in each ear), in order to prevent factors, such as child fatigue, to impact on the results^16^. Each test-band consisted of approximately 30 white noise segments of 6 seconds of duration each. Within the white noise segments there were numerous gaps in different positions and with variable durations, between 2, 3, 4, 5, 6, 8, 10, 12, 15 and 20 ms. Each one of these gaps appears six times per test band, making up a total of 60 gap presentations per list, and some stimuli did not have a gap inserted.

The children were told that they would hear a noise, and within that noise there were “pauses” or “moments of silence”, and whenever they perceived these silence intervals, they should respond with a gesture. We calculated the gap detection threshold (the shortest gap perceived by the patient in at least 66.6% of the times it was presented, that is, four times in six) and the percentage of correct answers by test-band (how many gaps were perceived in total)^17^.

The statistical analysis was carried out by means of the Minitab^®^ 16, Office Excel 2010^®^ and SPSS^®^ version 17 software. We used a 0.05 (5%) level of significance marked by an asterisk (*) in the results. The Kolmogorov-Smirnov normality test was employed, and it showed that the data sample has a normal distribution, allowing the use of parametric statistical tests.

## RESULTS

**Graph 1:**
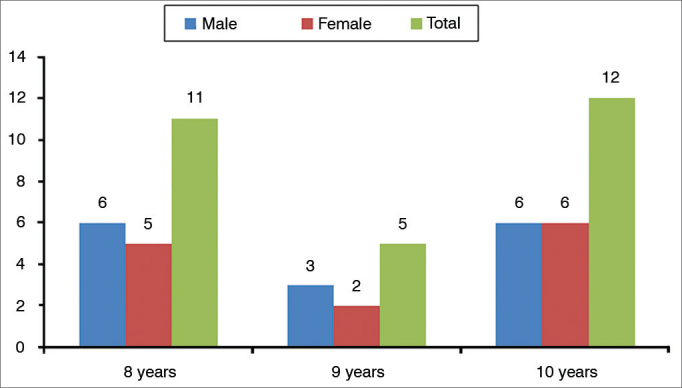
Sample characterization according to gender and age.

The studied sample was made up of 28 school-aged children, in the age range of 8 to 10 years (mean age of 9.04 ± 0.34 years). Graph 1 depicts the individuals’ characterization according to the three age groups and genders; and Table 1 shows the relative frequency distribution of males and females. The statistical analysis showed that although there was a higher percentage of males in the sample, 53.6%, there were no statistically significant differences vis-à-vis the females: 46.4%.

**Table 1:** Distribution of the relative frequency (percentages) for males and females.

Gender	N	%	*p*-value
Females	13	46.4%	
Males	15	53.6%	0.593

Two proportions equality test.

Table 2 depicts the results of the mean gap detection thresholds in the RGDT test, per frequency. Although there are differences in the mean values per tested frequency, the statistical analysis did not show significant differences (*p* = 0.4).

**Table 2:** Silence intervals threshold values in the RGDT test (ms) by frequency in the studied sample (N = 28).

RGDT	500 Hz	1 KHz	2 KHz	4 KHz	Final average
Mean	8.82	9.14	8.32	10.71	9.25
Standard deviation	4.30	5.68	6.09	5.73	3.67
CI	1.59	2.10	2.26	2.12	1.36
*p*-value			0.400		

ANOVA test with repetitive measures. CI: Confidence interval.

Table 3 shows the results of the gap detection thresholds and the percentages of correct answers in the GIN test, vis-à-vis the right and left ears. The statistical analyses, carried through by the paired *t-Student* test showed that there was no statistically significant mean difference between the ears in the GIN test, both in relation to the gap detection threshold as well as the mean percentage of correct answers.

**Table 3:** Gap detection thresholds (ms) and mean percentage of correct answers in the GIN test, in the comparison between right and left ear (N = 28).

GIN	Threshold		% Correct answers	
	RE	LE	RE	LE
Mean	4.32	4.43	73.3	74.6
Standard deviation	0.61	0.79	6.0	8.1
CI	0.23	0.29	2.2	3.0
*p*-value	0.449		0.261	

Paired *t-Student* Test. CI: Confidence interval.

Table 4 presents the results by evaluated frequency in the RGDT and the gap detection thresholds, and the percentage of correct answers per ear in the GIN test vis-à-vis males and females. The statistical analysis carried out concluded that there was no statistically significant difference between the genders, in the RGDT or the GIN tests.

**Table 4:** Results from the GIN test by evaluated ear, and RGDT by evaluated frequency, in relation to gender.

GIN		N	Mean	SD	*p*-value	RGDT		N	Mean	SD	*p*-value
RE threshold	Fem	13	4.38	0.65	0.62	500 Hz	Fem	13	10.15	5.51	0.13
	Male	15	4.27	0.59			Male	15	7.67	2.58	

LE threshold	Fem	13	4.38	0.87	0.79	1 KHz	Fem	13	9.31	6.2	0.889
	Male	15	4.47	0.74			Male	15	9	5.41	

% RE	Fem	13	73.7	5.8	0.751	2 KHz	Fem	13	7.77	5.42	0.664
	Male	15	73	6.3			Male	15	8.8	6.77	

% LE	Fem	13	74.5	9.5	0.92	4 KHz	Fem	13	10.77	4.94	0.963
	Male	15	74.8	7			Male	15	10.67	6.51	

ANOVA test.

With regards to the age variable, we used the Pearson's correlation to measure the degree of association between age and the GIN and RGDT results. To validate such correlations, we used the correlations test, and the results are presented on Table 5, in function of the *p*-value found. The statistical analysis showed no correlations between the GIN and RGDT tests pertaining to the age variable and is, therefore, considered a statistically independent variable.

**Table 5:** Correlation between the Age variable and the mean results from the GIN and RGDT.

	Age	
	Corr	*p*-value
RE GIN threshold	11.0%	0.577
LE GIN threshold	-12.3%	0.531
Mean RGDT	-29.8%	0.124

Correlation test/Pearson's Correlation.

And finally, we compared the mean value of the RGDT frequencies with the GIN gap detection thresholds in each one of the ears, and the results can be found on Table 6. By means of the statistical analysis carried through, it was possible to conclude that the mean value of the RGDT frequencies of 9.25 ms is considered statistically different from the mean values of the GIN test in each ear - 4.32 ms in the right ear and 4.43 ms in the left ear.

**Table 6:** Comparing RGDT frequency mean values and GIN thresholds per ear in the sample studied (N = 28).

Comparison	Mean RGDT	RE GIN threshold	LE GIN threshold
Mean	9.25	4.32	4.43
Standard deviation	3.67	0.61	0.79
IC	1.36	0.23	0.29
*p*-value		< 0.001*	

Paired *t-Student* test; CI: Confidence interval.

## DISCUSSION

This study involved the use of two TR tests in school-aged children aimed at checking the influence of variables such as gender, age range and right and left ears in the results from each test, as well as the analyses of the differences between the two assessment procedures from the clinical point of view.

With regards to the results presented, we did not find statistically significant differences between males and females, in both GIN and RGDT tests (Table 4). This data is in accordance with those from other papers, which also did not find influence of the gender in the gap detection threshold test^9^, ^10^, ^17^, ^18^.

Zaidan et al.^8^ compared the performance of normal young adults in RGDT and GIN, aiming at analyzing the differences between the two tests. 25 normal-hearing colleges students were evaluated - 11 men and 14 women. In their results, the authors found a statistically significant difference between the genders in both the tests, and males had better performance in the RGDT and the GIN tests. Such result does not corroborate the present study, but it is in agreement with the study carried out with the GIN test in Brazilian adults^16^. In the study carried out by Zaidan et al.^8^, the authors argue for a possible bias in their results that could justify the findings, since the male participants were music-therapy students and the women were not. We know that the better TR skill of musicians is due to their greater stimulation of auditory areas^19^.

Chermack & Lee^10^ compared the performance of 10 children of 7 to 11 years of age, without auditory and/ or learning complaints, in four TR tests: Auditory Fusion Test-revised (AFTR), Binaural Fusion Test (BFT), GIN and RGDT. In the RGDT test, the gap detection threshold mean value was 4.77 ms (± 1.83) and 4.6 ms in the GIN (± 1.07) for the right ear and 4.9 ms (± 0.99) for the left ear. Such values are in accordance with the findings of this study in the GIN test (4.32 ms ± 0.61 in the right ear and 4.43 ms ± 0.79 in the left ear); however, the mean RGDT value in the present study was 9.25 ms ± 3.67, higher when compared with the North American children's performances, but still within the normality values as per proposed by Keiths^20^ (Tables 2 and 3).

In this same cited study, the authors also presented values for the RGDT test carried through in another modality, by means of the “click” stimulus instead of the pure tone, and the gap detection mean value was 8.4 ms (± 5.25), closer to the values hereby presented. We found only one study in the searched literature that had the objective of comparing the RGDT versions with pure-tone-type of stimuli and click; however, in young Brazilian adults. 40 individuals with ages between 18 and 25 years were evaluated, and the authors found no statistically significant differences as to the temporal threshold for pure tones and clicks, and the mean values were 6.72 ms for pure tone and 6.43 ms for clicks^21^. We found no papers in the specialized literature discussing this difference in children; and except for the study by Chermack & Lee^10^, all others used RGDT with pure tone only, and found values higher than 4.77 ms, corroborating our findings. (9.25 ms ± 3.67).

Despite the divergence vis-à-vis the study with American children, the results of this study are within the normality values proposed by the authors of the RGDT and GIN tests, and values found in other studies carried out with children^5^, ^10^, ^20^, ^22^.

The findings differ from those by Balen et al.^9^, which evaluated the TR of 14 school-aged children in the age range of 6 and 14 years, by means of the RGDT and GIN tests, aiming at comparing the two procedures. The RGDT (10.50 ms ± 5.28) and GIN (5.7 ms ± 2.87 right ear and 5.4 ms ± 1.07 left ear) results were slightly higher (worse) than the values found in the present study, however in accordance with the study carried out in school-aged children from the city of Recife^23^.

One of the hypotheses that could justify such discrepancies, besides differences in the studied age ranges, is the reduced number of evaluated individuals, since our sample was larger and more homogeneous (Graphic 1 and Table 1). With regards of the age range, the study from Balen et al.^9^ evaluated school-aged children between 6 and 14 years, while the present study evaluated school-aged children between 8 and 10 years. Recent research regarding the neuromaturation course of the TR skill pointed to the fact that this skill develops by 7 years of age, and children in the age range between 6 and 7 years can present higher thresholds in comparison with other age ranges, justifying the differences found^3^, ^24^.

In agreement with the study, the GIN test author points out that there are but a few maturity influences in TR tests after 7 years of age^7^, the results presented here did not show age influence on the GIN and RGDT tests (Table 5). Other studies have also corroborated this data^3^, ^4^, ^18^, ^22^.

We did not find significant differences in relation to the evaluated frequencies in the RGDT (Table 2). The mean results point to a slightly higher value in the 4000 Hz frequency. We found only one study reporting a significant difference in relation to the 4000 Hz frequency, which also had a higher value when compared to the other frequencies^23^.

Specifically related to the GIN test, we did not find differences between the right and left ears vis-à-vis the gap detection thresholds and total percentages of correct answers (Table 3), and such result was also not corroborated by other aforementioned studies^8^, ^9^, ^10^, ^18^, ^22^.

Although some authors report a right ear advantage (left hemisphere) in tasks that require the TR skill^25^, the results are in agreement with the statement from Baran & Musiek^26^, that monotic tests are useful to detect deficits, but they can “mask” the left hemisphere dominance in these tasks, since both ipsi and contralateral pathways are activated, resulting in a similar performance from both the ears in the test.

In the comparative statistical analysis of the results from both tests, we found differences in the performance of our sample, and the gap detection thresholds in the RGDT test were significantly higher than the ones obtained in the GIN test in each ear (*p* < 0.001) (Table 6).

Although both tests properly classified the children evaluated as normal in their TR skill, from the clinical point of view, the differences found are extremely relevant, since such result seems to happen due the differences in the parameters of each procedure and it can influence the examiner's decision to use one test or the other^8^.

In the study published by Balen et al.^9^, the authors also found this difference in the performance of children in the two tests; however, because of the small number of individuals evaluated (n = 14), and the results from this finding were presented only in a descriptive fashion, we consider the importance of our study - a larger number of children was assessed, and differences were statistically proven.

In the above-mentioned paper, the authors raise the hypothesis that the GIN and the RGDT tests are not evaluating the same auditory skill, or they require non-auditory processes in the requested tasks. Such hypothesis is based on the statement from the author of the GIN test, stating that the RGDT is, in fact, an auditory fusion test^17^.

By definition, the gap detection requires the individual to hear stimuli which have varied interstimuli intervals, and report the perception of a break-gap, and threshold found represents the shortest perceived gap. The so-called binaural fusion tasks require the evaluated individual to report when two stimuli are perceived as only one, and the threshold found represents the shortest silence interval between two sounds that the individual can detect, not allowing the fusion of the two stimuli^27^.

Chermack & Lee^10^ stated that clinically, binaural fusion and detection tasks are commonly used to describe the same skill, despite the neurological evidence that different neurophysiological processes are required in each one of them.

From this, one of the hypotheses that can be raised with regards to RGDT test resulting in higher thresholds when compared with the GIN in the same population, is that the RGDT test parameters require the individual to perform a more complex auditory task, one involving auditory fusion (at the time when the two stimuli are perceived as one single sound) and the temporal resolution (at the time when the gap is detected). On the other hand, the GIN, could be considered a test of temporal resolution only.

The RGDT has been described in the specialized literature as an easy and fast test, especially when compared with the GIN - which requires longer execution times. However, despite this advantage, the differences previously discussed with regards to the nature of the task that is being requested seem to also influence the complexity of individual's response in the test. The RGDT requires a more complex response than the GIN, since the patient is instructed to say or use gestures do show that he/she perceived one or two stimuli. On the other hand, the GIN test requires a simpler response, one considered non-verbal, in which all the patient has to do is signal when he/she perceived a break in the stimulus; thus making it easier to assess different populations, including those individuals who find it difficult to respond in tests based on speech and language^10^.

In our clinical practice, during the tests application, we noticed that some children had difficulties in understanding the requested task in the RGDT test, and this made it necessary to introduce an expanded version of the test, with longer intervals between the pairs of pure tones, so that the task could be properly carried through. Since it had not been the objective of this study, these children were taken off the sample due to their erratic answers. But we know that it is common to have this inconsistency in the application of the RGDT which can be, to a large extent, related with the factors argued in relation to the complexity of the evaluated task and the requested reply here to be cognitively more elaborated in relation to the GIN parameters.

Other factors also seem to influence such differences and deserve to be argued. On the RGDT, the method used to establish the gap detection threshold is based on “yes”/”no” answers - since the patient will report if “one” or “two” sounds were perceived, having 50% of possibility to be right or wrong on the answer^16^. From the statistical point of view, there is great likelihood of having chance play its part when the patient is distracted, or even when he/she has a wrong perception of that presentation -because the test is based on only nine presentations of different gap durations.

In the GIN test, chance and false-positive results may be considered as less important, because each gap appears six times in each test-band, and the threshold is considered as the one the patient perceived four out of six times - as long as there has been uniformity vis-à-vis the other following gaps of longer duration. That is, if the patient perceived the 4 ms gap four times, but the 5 ms gap only three times, there is a high possibility that it had been carelessness or chance, and the next value in which the consistency of at least four out of six correct answers is repeated will be considered the threshold.

Samelli^28^ stresses other advantages in the parameters used in the GIN test, which can be compared to the RGDT test, such as the use of gaps inserted in white noise in the GIN and pure tones in the RGDT, and the GIN ability to analyze separately the auditory canals, differently from the RGDT, which is binaural.

The white noise causes the activation of numerous auditory canals simultaneously, enabling the stimulation of higher levels in the auditory pathway, different from what happens in the stimulation by pure tone, which evaluates small portions of the auditory pathway, besides providing spectral clues that can distort the temporal task evaluation^28^.

Finally, from the point of view of our clinical practice, despite the advantages hereby discussed about GIN parameters compared to those from the RGDT, the GIN test duration time is a relevant factor taken into account when choosing the tests that will make up the auditory processing assessment array of tests. In our study, the mean time for GIN testing was about 15 to 20 minutes, being considered by some of the children as tiring - which was also evidenced in the study by Balen et al.^9^. The RGDT, is of fast and easy deployment, as long as the evaluated individual has properly understood the task requested.

Despite the GIN test advantages in assessing the TR skill, fatigue can negatively influence the child's performance, and the examiner must consider the time of execution for each procedure, and this has been a determining factor when the examiner is to choose the test to be employed.

Thus, we find it feasible to consider whether it is possible to reduce the number of segments presented by test-band in the GIN, so that they may have more than three gaps each, or even check whether there is indeed a need for so many gaps lasting much longer than expected, as the 15 and 20 ms, since in practical terms these gaps are easily detected, even by those with difficulties and altered thresholds around 8, 10 and 12 ms.

Having all of the above regarding the GIN advantages vis-à-vis the RGDT in TR assessment, this study makes us think about the possibility of developing a reduced version of the GIN test, without compromising the parameters hereby discussed, reducing only the test duration time. A reduced version would need to involve new studies, both in normal adult and pediatric populations, as well as for populations with different complaints. We believe that, this will make the GIN better accepted and truly included in the complete test array used in auditory processing assessment.

## CONCLUSION

We did not find statistically significant differences in relation to the gender and age range variables both in the RGDT and in the GIN, and there was no significant difference between the frequencies in the RGDT test and between the right and left ears in the GIN. The mean gap detection threshold found in the RGDT test was 9.25 ms ± 3.67, and 4.32 ± 0.61 in the right ear and 4.43 ms ± 0.79 in the left ear of the GIN test.

The statistical analysis pointed to a statistically significant difference vis-à-vis the sample performance in the two tests, and the mean GIN gap detection threshold for each ear was lower than that of the RGDT test, in both genders.

Significant differences in relation to the nature of the requested task, type of response, stimulus employed and presentation duration were discussed in order to understand discrepancies found in the results, and we stressed the GIN advantages as a TR assessment procedure in comparison with the RGDT. We highlight the importance of creating a version of the GIN requiring shorter testing time.
